# Supplementary material for: High endemicity of Q fever in French Guiana: A cross sectional study (2007–2017)
Source: PLoS Negl Trop Dis. 2022 May 18;16(5):e0010349. doi: 10.1371/journal.pntd.0010349 (PMC9197051; doi:10.1371/journal.pntd.0010349)
Supplement: Attachment — Submitted filename: Response.docx [file pntd.0010349.s001.docx]

Cayenne, June 23^rd^ 2021

I am sorry for the delay due to the computer problems we had the opportunity to discuss.

The wave of covid19 that French Guiana is currently undergoing and in which I am very involved, prevented me from being very reactive once I received your answers.

The following section has been added.

Sincerely yours

Loïc Epelboin, on behalf the co-authors

Ethic Statement

As authorized by French law, we based this study on the reference methodology MR-004, which governs the processing of personal data for the purposes of study, evaluation or research not involving the human person. This is research involving the human person and concerning the re-use of data. Individual information: Non-opposition was sought via a telephone call based on the contact information found in the patients' medical records. Patients were informed of the study and its objectives and of their right to refuse to participate in the research or to withdraw at any time without any prejudice and without having to justify the reasons for their decision. For those patients for whom no contact was made during the first attempt, a second attempt was made at different days and times from the first call. For patients for whom neither telephone contact was successful, an information note was sent to the patient's mailing address. Collective information: a collective information note was present in the departments concerned, published on the hospital website and included in the hospital's welcome booklet.
