# Supplementary material for: High endemicity of Q fever in French Guiana: A cross sectional study (2007–2017)
Source: PLoS Negl Trop Dis. 2022 May 18;16(5):e0010349. doi: 10.1371/journal.pntd.0010349 (PMC9197051; doi:10.1371/journal.pntd.0010349)
Supplement: Attachment — Submitted filename: Reviewer v2.0.docx [file pntd.0010349.s002.docx]

Reviewer's Responses to Questions


**Q1 - Reviewer #1: while important background information is given in the introduction certain elements are missing. There should be a brief description of the medical resources available in French Guiana - how many doctors?**

A1 : The following sentence was added to the MS : “The healthcare offer in French Guiana is divided between 3 public hospitals located in the three main cities of the territory (Cayenne, Kourou and Saint Laurent du Maroni), as well as 18 health centers spread throughout the territory, including in the most remote areas.In 2020, 648 physicians were registered with the Departmental Council of the Order of Physicians of French Guiana, of which 583 were active.”And the following reference was added : *Atlas de la démographie médicale en France - Situation au 1er janvier 2021. Ordre National des Médecins Conseil National de l'Ordre. 2021 Pages 141.*

**Q2 -Has the health department informed doctors about the high rate of Q fever in FG in the past?**

A2 - No, there is no specific information about Q fever available to physicians in the territory from the Health Authorities.

**Q3 - How many serum samples are tested each year in FG - give the number and percent positive per year.**

A3 - Unfortunately, although the question is very interesting, we are unable to answer this question. Indeed, the samples were taken in 4 different laboratories, the polyvalent laboratory of the Institut Pasteur of French Guiana, the national reference center for Q fever located at the InstitutHospitalo-Universitaire of Marseille, and 2 private laboratories located in mainland France Cerba and Biomnis. Several of them answered that they had changed their software since then and were not able to provide us with this information for the requested period. In addition, patients may have had several samples taken in different laboratories during their follow-up, which makes it difficult to obtain a reliable positivity rate.

**Q4 - Biominis or Cerba Central labs -- where are they located.**

A4 - The Biomnis laboratory is located in Ivry-sur-Seine and the Cerba laboratory in Saint-Ouen-l'Aumône, both in the Paris suburbs. This was added in the MS.

**Q5 - Did all 4 laboratories that performed tests for C. burnetii use the same materials - eg did one supplier provide the materials to all 4 labs?**

A5 – No, the 4 laboratories were independent of each other, so they did not use the same materials and the suppliers were different.

**Q6 - Did all 4 labs use the same criteria for a positive test.**

A6 - Conversely, all 4 laboratories used the same criteria for a positive test, criteria that were issued by the CNR of Q fever in Marseille. This was added in the MS.

**Q7 - Fishers test -- ??Fishers test**

A7 – This has been modified.

Reviewer #2: (No Response)

Reviewer #3:

**Q8 - Cite the references showing that the criteria for serodiagnosis of Q fever are appropriate: Persistent Q fever generally has higher antibody titers to Phase I than to Phase II.**

A8 – The details of the serodiagnosis are well described in “Eldin C, Melenotte C, Mediannikov O, Ghigo E, Million M, Edouard S, Mege JL, Maurin M, Raoult D. From Q Fever to Coxiella burnetii Infection: a Paradigm Change. Clin Microbiol Rev. 2017;30(1):115-90.” And “Tissot-Dupont H, Thirion X, Raoult D. Q fever serology: cutoff determination for microimmunofluorescence. Clin Diagn Lab Immunol 1994; 1:189–96.” These references have been inserted.

**Q9 - Describe in more detail the epidemiological items and risk factors investigated.**

A9 – This was done. The following sentence was inserted “Medical records were reviewed to confirm if the disease was acute or persistent. Demographic data (gender, age, place of residence (town and neighborhood) and country of birth) were collected retrospectively. The other data that were collected were : dry or wet season, clinical form (acute or persistent), main clinical picture of acute Q fever (isolated fever, pneumonia, myocarditis, thrombo-embolic event) or persistent (endocarditis, vascular infection).”

**Q10 - Even if the analysis is based on anonymization, the ethical response is insufficient. It should be stated that the research plan has been approved by an authorized committee. The method of obtaining information from the subject by telephone may be inappropriate in many cases as informed consent.**

A10 – the ethical aspects session was completed, as following :

This is a non-interventional study of retrospective data collected only in the context of routine care. The data were collected from a single center, the Groupement Hospitalier de Guyane, which includes the 3 coastal hospital centers (Saint Laurent du Maroni, Kourou, and Cayenne) and the 17 health centers of the remote area of French Guiana, that are administratively dependent on the Cayenne Hospital Center. Some patients have been diagnosed by a general practitioner, but were then referred to the specialized consultation at the hospital of Cayenne. Data collection in the medical record was done pseudonymously, for all patients diagnosed with Q fever between 2007 and 2017. Only the information strictly necessary for the treatment and purpose of the research was collected and these data kept for the duration of the study and then archived for 15 years. The data were identified by a code and the patients' initials. Finally, the data were collected from data already collected previously from medical records. This research was in compliance with the law "Informatique et Libertés" of January 6, 1978 as amended and the law No. 2018-493 of June 20, 2018 on the protection of personal data and Regulation (EU) 2016/679 of the European Parliament and of the Council of April 27, 2016 on the protection of individuals with regard to the processing of personal data and on the free movement of such data (RGPD). The data was transferred and collected in accordance with the reference methodology MR004 of the Commission Nationale de l'Informatique et des Libertés (CNIL) for which the Centre Hospitalier de Cayenne has signed a compliance commitment.

--------------------


Reviewer #1:

**Q11 - You indicated that you would exclude patients if data were missing - the results do not indicate that any patients were excluded.**

A11 – A flow chart was built, and the excluded patients are indicated.

**Q12 - It is important to give the percentage of serum samples that were positive each year**
A12 – We looked for this results that were not easily available. SO we add this paragraph in the Method section

:
*Calculation of the seropositivity rate*

In a second step, in 2022, we sought to calculate the rate of *C. burnetii* serology positivity. We therefore contacted the 4 laboratories where the samples came from to find out the number of patients tested for Q fever during the study period. The total number of patients in the study with a diagnosis of Q fever was calculated by dividing the number of patients tested for Q fever by the number of patients with a non-zero IgM and/or IgG titre against *C. burnetii.*

*And this one in the result section*

*Calculation of the positivity rate*

For the calculation of the positivity rate, we secondarily contacted the 4 laboratories. This was not easy, because for several of them, the software had changed, and the number of tests performed over the study period as well as the number of patients having received a diagnostic test for C. burnetii could not be obtained from all of them, and over the entire study period (Table 3).

Thus, the number of patients with non-zero C. burnetii serology over the study period was 17.4% (N= 2426/11701) for the laboratory of the Institut Pasteur in French Guiana over the period 2007-2017, 66.1% (N=148/224) for the national reference center in Marseille over the period 2014-2017, and 8.2% (N=71/869) for the Cerba laboratory. Data could not be obtained retrospectively for the Biomnis laboratory, but a very limited number of tests were sent to them over the period. Finally, if we relate the number of patients with a diagnosis of Q fever (acute or chronic) over the study period to the number of patients tested, we find a positivity rate of 5.6% and a number of patients with a non-zero C. burnetii serodiagnosis related to the total number of patients tested over the study period of 27.8% (695/2,499).

**Q13 - Of the 681 patients with acute Q fever - 513 had pneumonia and  22 others had a variety of illnesses - what about the 46 others??**

A13 – Thanks to your pertinent comment, we were forced to go back into the clinical records, which allowed us to restore the number of cases studied. Indeed, among the 700 cases initially identified as acute forms, we realized that they were patients with another pathology and an incidental finding of a serological sequela of Q fever. Some cases initially labeled as persistent Q fever were also reclassified after reopening the medical records. Finally, we were able to reclassify all the clinical pictures of acute and persistent form that are presented in the flow chart.

**Q14 - After describing Method in more detail, show considerations in terms of race and other indicators of genetic background rather than country of birth.**

A14 - The European regulation prohibits the collection or use of "sensitive data", which includes "alleged racial or ethnic origin, political opinions, religious or philosophical beliefs or trade union membership, as well as the processing of genetic data, biometric data for the purpose of uniquely identifying a natural person, data concerning health or data concerning the sex life or sexual orientation of a natural person”. (Source : <https://www.cnil.fr/fr/definition/donnee-sensible>) For this reason, we have retained only the country of birth, as other information cannot be collected.

Q15 - **The median age should be shown by gender as well as overall.**

A15 – The median ages were calculated by gender, and this sentence was added : “The median age was 45.3 years (IQR: 33.7-56.5) 45.3 (34.5-55.8) for men and 45.2 (33.2-57.9) and the majority (65.9%) belonged to the age groups between 30 to 59 years. “

**Q16 - For each city or region that you have categorized, show the ratio of the population by industry and the rate of positivity among them.**

A16 – We built a table with these informations, table 1, but not the data about indudtry, that we were not able to find.

--------------------


Reviewer #1:

**Q17 - You indicate that there is some uncertainty as to the source of Q fever in FG. References 20 and 21 are highly suggestive that the three toed sloth and Capybara play a role. Can you expand on the reason for your uncertainty.**

A17 - It is difficult to say what exact role these 2 species play in the epidemic cycle of Q fever. Indeed, only one three-toed sloth has been found positive, and this in the particular situation of a localized cluster potentially related to this sloth. Nevertheless, a dozen other sloths were subsequently tested all negative. Moreover, most of the Q fever patients interviewed in daily clinical practice report absolutely no contact with a sloth. The same is true for the capybara, which is rarely encountered in peri-urban areas, and of which only 1 individual has been found positive so far. Studies are currently underway to better understand the extent of the C. burnetii reservoir in wildlife.

Reviewer #3:

**Q18 - Conclusion is simple and clear. However, Methods and Results are not enough.
So, this is a weak basis for consideration.**

A18 – The methods and results have been widely completed.

--------------------

<b>Editorial and Data Presentation Modifications?</b></br><br/>
Use  this section for editorial suggestions as well as relatively minor modifications of existing data that would enhance clarity. If the only modifications needed are minor and/or editorial, you may wish to recommend “Minor Revision” or “Accept”.

Reviewer #1: (No Response)

Reviewer #2: (No Response)

Reviewer #3: No

--------------------

<b>Summary and General Comments</b></br></br>
Use  this section to provide overall comments, discuss strengths/weaknesses of the study, novelty, significance, general execution and scholarship. You may also include additional comments for the author, including concerns about dual publication, research ethics, or publication ethics. If requesting major revision, please articulate the new experiments that are needed.

**Q19 - Reviewer #1: This is an important paper**

A19 -

Reviewer #2: The authors concluded that the incidence of Q fever in French Guiana remained high and stable and the highest in the world. They also found that the risk factors associated with Q fever were male gender, being born in mainland France with age between 30 to 59 years-old and a residence in Cayenne and surroundings.

**Q20 - The description of method is too simple and rough. What is the method for detecting antibodies in patients with Q fever? Is it a commercial kit or a homemade one? How specific is this reagent? When these problems are not clear, it is impossible to judge whether these patients really have Q fever.**

A20 – The method section has been completed

**Q21 - The author mentioned PCR in the method. What are the primers and protocol of the PCR?**

A21 – As there is no case positive with PCR, this sentence ahas been removed.

**Q22 - Is any patient PCR positive?**

A22 – No (cf supra)

**Q23 - What evidence to suppot the claim that the incidence of Q fever in French Guiana is the highest in the world**.

A23 – The review of the literature let us think that there is no territory identified currently with such a stable incidence rate.

**Q24 - In addition, misspellings such as acutez Q fever and inonly in hte manuscript need to be corrected.**

A24 – The MS has been entirely revied.

Reviewer #3:

Q25 - Updating epidemiological information in endemic areas with high Q fever is extremely important and meaningful. However, the description of Methods and Results is insufficient, and many of the discussions are based on multiple unpublished data. I would like to see the presentation of more detailed information and a discussion that eliminates unpublished data as much as possible.

A25 – The description of the methods and results has been widely completed, and some figures and tables added.

Q26 - Also carefully present climatic and geographical information, even if it is a secondary objective.　Only a small percentage of readers of international journals are familiar with French Guiana.

A26 – We added this sentence: The cycle of seasons in French Guiana is punctuated by the variations in latitude of the Inter Tropical Convergence Zone which determines 4 seasons: small rainy season from December to mid-February. Small summer of March between mid-February and the end of March (very variable from one year to another). Big rainy season in April, May and June. Tropical wave period in July and August (intermediate period between the great rainy season and the dry season). Dry season: from September to the end of November.

--------------------

PLOS authors have the option to publish the peer review history of their article (<a href="<https://journals.plos.org/plosntds/s/editorial-and-peer-review-process#loc-peer-review-history>" target="_blank">what does this mean?</a>). If published, this will include your full peer review and any attached files.<br><br>

If you choose “no”, your identity will remain anonymous but your review may still be made public.<br><br>

<b>Do you want your identity to be public for this peer review?</b> For information about this choice, including consent withdrawal, please see our <a href="<https://www.plos.org/privacy-policy>" target="_blank">Privacy Policy</a>.

Reviewer #1: Yes: Thomas J. Marrie MD

Reviewer #2: No

Reviewer #3: No


Figure Files:
While revising your submission, please upload your figure files to the Preflight Analysis and Conversion Engine (PACE) digital diagnostic tool, [https://pacev2.apexcovantage.com](https://pacev2.apexcovantage.com/). PACE helps ensure that figures meet PLOS requirements. To use PACE, you must first register as a user. Then, login and navigate to the UPLOAD tab, where you will find detailed instructions on how to use the tool. If you encounter any issues or have any questions when using PACE, please email us at figures@plos.org.
 
Data Requirements:
Please note that, as a condition of publication, PLOS' data policy requires that you make available all data used to draw the conclusions outlined in your manuscript. Data must be deposited in an appropriate repository, included within the body of the manuscript, or uploaded as supporting information. This includes all numerical values that were used to generate graphs, histograms etc.. For an example see here: <http://www.plosbiology.org/article/info%3Adoi%2F10.1371%2Fjournal.pbio.1001908#s5>.
 
Reproducibility:
To enhance the reproducibility of your results, we recommend that you deposit your laboratory protocols in protocols.io, where a protocol can be assigned its own identifier (DOI) such that it can be cited independently in the future. Additionally, PLOS ONE offers an option to publish peer-reviewed clinical study protocols. Read more information on sharing protocols at <https://plos.org/protocols?utm_medium=editorial-email&utm_source=authorletters&utm_campaign=protocols>

__________________________________________________
In compliance with data protection regulations, you may request that we remove your personal registration details at any time.  (Use the following URL: <https://www.editorialmanager.com/pntd/login.asp?a=r)>. Please contact the publication office if you have any questions.
